# Biogeographic implications of plant stature and microclimate in cold regions

**DOI:** 10.1038/s42003-023-05032-5

**Published:** 2023-06-26

**Authors:** Christian Körner, Alex Fajardo, Erika Hiltbrunner

**Affiliations:** 1 Department of Environmental Sciences, University of Basel, Schönbeinstrasse 6, 4056 Basel, Switzerland; 2 Instituto de Investigación Interdisciplinaria (I3), Vicerrectoría Académica, Universidad de Talca, Avenida Lircay s/n, Talca, 3460000 Chile

**Keywords:** Plant ecology, Plant sciences, Ecophysiology

**arising from** A. Crivellaro et al. *Communications Biology* 10.1038/s42003-022-03732-y (2022)

All species and life forms of plants reach a low temperature range limit either related to their freezing tolerance or their capability to develop and grow^1–3^. Because small alpine herbs are less lignified than trees, Crivellaro et al. (CA)^4^ assume a critical role of lignification for explaining the low temperature treeline. We find that their analysis does not support such an inference. Herbs simply represent a larger species fraction at high elevation because of the microclimatic advantage of small stature in cold climates, whereas trees experience the full strength of atmospheric circulation.

**Fig. 1:**
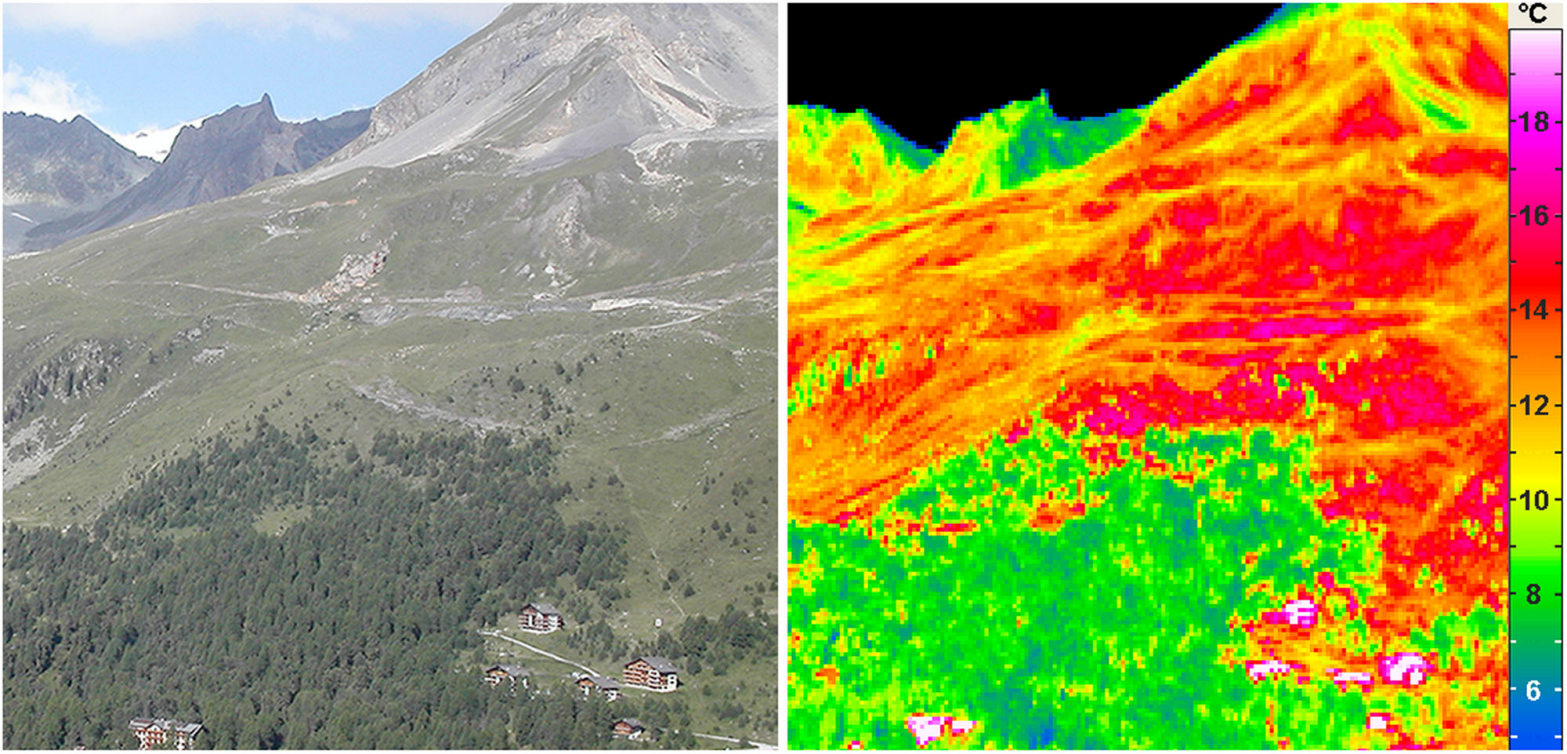
Moving from treeline to alpine, means moving from cool to warm. As plant stature shifts at treeline from tree size (green, *Pinus cembra* in the lower part of the Figure, c. 8 °C) to alpine grassland (red, c. 15 °C), the conditions experienced by plants become significantly warmer when the sun is out^1,2^. This infrared thermal image taken at c. 11:00 a.m. at Arolla, Swiss central Alps (46° 01’ 31” N, 7° 28’ 24”E, tree limit at 2200 m) illustrates why high elevation selects for small stature plants, and why air temperature is unsuitable to infer plant temperature above treeline^1,2,8,9^ (modified from Ref. ^2^).

Capitalizing on double-stained microscopic cross-sections of stems of 1770 species, mostly collected by Fritz Schweingruber, CA use the lignified (woody) red area fraction (CA’s DCWL, degree of cell wall lignification) of these sections to infer stem strength and hydraulic capacity. The work builds upon a sister paper referring to the same data, in which insufficient cell wall lignification is supposed to hinder alpine and arctic plants in growing tall^5^. From that, CA further infer a potential restriction of the life form tree in cold climates (CA’s Fig. 1). However, (a) their data (Crivellaro et al.’s Fig. 6) show a high abundance of poorly lignified herbs also at low elevation, underlining the life-form and the species-specific nature of that trait, (b) CA did not find reduced lignification in shrubs and trees at higher elevation (Crivellaro et al.’s Fig. 5c), nor (c) did their data include any trees at or near treeline (Crivellaro et al. ‘s Suppl. Data 1). Using WorldClim^6^ climate data for 1–10 additional 2.5’ grid cells for which the species had been reported by GBIF^7^ but was not sampled, the one sample’s traits (red area fraction and plant height) were paired with these climatic data. Neither were selection criteria for the extra sites described, nor were differences in season length accounted for. This way, CA arrive at 13028 species trait-climate combinations, with across-species correlations often significant due to the pseudo-replication of the independent variable (the red area fraction).

From the increasing abundance of small herb species at high elevation, CA infer that less lignified small plants are often found in cold climates, and that cell wall lignification represents a biochemical bottleneck in the cold. Given that herb species with poor lignification are also abundant at low elevation (Crivellaro et al.’s Fig. 6; point 3 below), and the lack of replication per species across thermal gradients, such a low temperature effect on lignification remains unsubstantiated. Small and herbaceous plant species are selected for at high elevation owing to their rapid development (short growing season) and microclimatic benefits^2^. Whether phenotypic responses are involved, cannot be resolved with one-sample-per-species.

CA aimed at answering, (in their opinion) an unanswered question: “What is causing the low temperature range limit of tree species and the range limit of the tree life form?”—a quote from a paper by Christian Körner that summarized answers to that rhetorical question^1^. In brief, low temperature limits growth similarly in all cold adapted plants^7^, but trees are affected at lower elevation due to their aerodynamic coupling to air temperature^1^. The alpine environment selects for small stature plants, because these can escape the low temperature of the atmosphere by reducing aerodynamic heat exchange, and thus, warm under solar radiation (Fig. 1).

CA’s samples did not include gymnosperms (the most prominent treeline taxon) and data for angiosperm tree species largely came from warm places (Crivellaro et al.’s Suppl. Data 1). The final conclusion that understanding lignification will help in explaining treeline is not reflected by the facts presented. Assessing low-temperature related responses, would require sampling the same species across critically low temperatures. Below are five points that underpin our concerns. These relate to fundamental questions of what causes range limits of species and life forms of plants.

1. In contrast to the temperature of trees, which is close to air temperature (hardly any microclimate effect), air temperature is unsuitable to infer temperatures of small stature plants^1,2,8,9^ (Fig. 1). Employing air temperature, CA neglect the content of the microclimate literature they cite. Lack of data does never justify unfounded conclusions. A meaningful analysis could have used estimates of meteorological season length, and thus, account for the fact that short seasons select for small herbaceous species with fast seasonal tissue turnover, both, in hot and cold environments.
2. The correlations with air temperature from up to 10 extra sites of contrasting elevations from where no plants were sampled are ecologically questionable (Crivellaro et al.’s Suppl. Data 1). If climate had the suggested effect on lignification, why should these different climates correlate with the one sample’s traits?
3. The life form concept is central to plant biology and biogeography. CA undermine the function of plant morphology by pooling tall trees, shrubs and dwarf shrubs into one category (listed as ‘trubs’ in Crivellaro et al.’s Suppl. Data 1, because they are woody^10^), thus confusing morphology (stature) with tissue anatomy. Rooted in phylogeny, these plant architectures are selected for coping with disturbances (e.g. herbivory, fire), to trap nutrients (e.g., cushion plants), or to escape harsh atmospheric conditions by ‘engineering’ aerodynamic shelter and thus, warmth. Crivellaro et al.’s Fig. 4 pools all these morphotypes (life forms). The data for less than 1m plant height in Crivellaro et al.’s Fig. 6 largely overlap, causing the ‘blue’ (hardly-lignified) herb symbols from low elevations to become hidden under the red symbols for woody species.
4. *The red (lignified) fraction* of microscopic cross-sections amalgamates conduits and non-conduit tissue. This limits a functional interpretation^11^. The fractionation and arrangement of the various tissue types including parenchyma (blue fraction) is species-specific^12^. In our view, *lignified* is not synonymous to wood as CA present it.
5. Cell walls can lignify at constant 0 °C^13^. Other than quoted, xylem lignification in stalks of *Soldanella pusilla* (Primulaceae) also occurs under snow and not *after* snow melt. Assessed by Raman spectroscopy, conduit walls were lignified while growing at the 0 °C soil-snow interface. However, the peripheral sclerenchyma ring lignified after snow melt only. Otherwise, stalks could not bend upright during snowmelt as they do. CA take the sclerenchyma lignification immediately after melt-out as support of their lignin limitation hypothesis^4^, neglecting both, the ongoing lignification of xylem at 0 °C, and the fact that even the coldest place on earth with plants^14^ is warmer than constant 0 °C. In fact, these data illustrate that it requires only a few hours of sunshine to assemble monomers into lignin polymers in otherwise cold regions^13^. All of CA’s samples came from plants that did grow at much warmer conditions than constant 0 °C. In situ Peltier-cooling by -3 K (corresponding to a 550 m higher elevation) during xylogenesis at the treeline had no effect on tree ring lignification, but rose the risk of early season frost rings^15^.

In summary, CA’s study arrives at suggestions that are not supported by the data presented, conclusions are tied to spot findings of ‘blue rings’ in data from other sources that failed to stain red because of either freezing damage in spring or developmental mismatches in autumn as discussed by Körner et al.^15^. We do share CA’s valuation of such ephemeral blue rings in response to extreme events for validating tree ring chronologies. To us, the high elevation herb data (largely from Ladakh, India) are unsuitable to make a treeline case without assessing data for treeline and phenotypic low temperature responses of lignification.
